# Estimation of ALU Repetitive Elements in Plasma as a Cost-Effective Liquid Biopsy Tool for Disease Prognosis in Breast Cancer

**DOI:** 10.3390/cancers15041054

**Published:** 2023-02-07

**Authors:** Madhumathy G. Nair, Rakesh S. Ramesh, Chandrakala M. Naidu, Apoorva D. Mavatkar, Snijesh V. P., Vishakha Ramamurthy, Vidya M. Somashekaraiah, Anupama C. E., Kiruthiga Raghunathan, Anuradha Panigrahi, Manjula Das, Sujan K. Dhar, Jyothi S. Prabhu

**Affiliations:** 1 Division of Molecular Medicine, St. John’s Research Institute, St. John’s Medical College, Bangalore 560034, India; 2 Department of Surgical Oncology, St. John’s Medical College and Hospital, Bangalore 560034, India; 3 Molecular Immunology Program, MSMF, Narayana Health City, Bangalore 560099, India

**Keywords:** breast cancer, liquid biopsy, ctDNA, ALU 247, prognosis, disease progression

## Abstract

**Simple Summary:**

Liquid biopsy is now extensively utilized as companion diagnostics for screening, identifying actionable alterations in tumors, for monitoring therapeutic response and residual disease. We assessed cell-free DNA (cfDNA) as a liquid biopsy tool and probed its utility in breast cancer patients. Total cfDNA was extracted from the plasma of 167 breast cancer patients. Patients with larger fragments of cfDNA estimated through ALU 247 in their post-operative plasma, were associated with a significant poor disease-free survival. Derivation of a nomogram showed that the addition of ALU 247 with other conventional parameters significantly improved the ability for predicting prognosis. Our results confirm the utility of cfDNA as an evolving liquid biopsy tool for molecular evaluation.

**Abstract:**

Background: Liquid biopsy is widely recognized as an efficient diagnostic method in oncology for disease detection and monitoring. Though the examination of circulating tumor cells (CTC) is mostly implemented for the assessment of genomic aberrations, the need of complex methodologies for their detection has impeded its acceptance in low-resource settings. We evaluated cell-free DNA (cfDNA) as a liquid biopsy tool and investigated its utility in breast cancer patients. Methods: Total cell-free DNA was extracted from the plasma of breast cancer patients (n = 167) with a median follow-up of more than 5 years, at various stages of the disease. Quantitative PCR was performed to estimate the copy numbers of two fractions of ALU repetitive elements (ALU 115 and ALU 247), and DNA integrity (DI) was calculated as the ratio of ALU 247/115. Mutations in TP53 and PIK3CA in the cfDNA were estimated by next-gen sequencing (NGS) in a subset of samples. Associations of the levels of both the ALU fragments with various clinico-pathological factors and disease-free survival at various stages were examined. Nomogram models were constructed with clinical variables and ALU 247 levels to predict disease-free survival and the best performing model was evaluated by decision curve analysis. Results: DI and ALU 247 levels were significantly lower (*p* < 0.0001) in the post-operative plasma when compared to their pre-surgery levels. DI and ALU 247 were found to be significantly higher in patients with metastasis (*p* < 0.05). Patients with higher levels of ALU 247 in their post-operative plasma had significant poor disease-free survival (*p* = 0.005). Higher levels of ALU 247 in the circulation also correlated with low tumor-infiltrating lymphocytes (TIL) within their primary tumors in the ER-negative breast cancer subtype (*p* = 0.01). Cox proportional hazard analysis confirmed ALU 247 as an independent variable of disease-free survival both in univariate and multivariate analysis [HR 1.3 (95% CI 1.047 to 1.613, *p* = 0.017)]. The nomogram model showed that the addition of ALU 247 with other variables significantly improved (C-index 0.823) the predictive ability of the model. Conclusion: Our results confirm the utility of cfDNA as an evolving liquid biopsy tool for molecular analysis. Evaluation of larger fragments of cfDNA estimated through ALU 247 can provide vital information concurrent with the pathological process of disease evolution in breast cancer and warrants expansion to other cancer types.

## 1. Introduction

The evolution of targeted therapies and advanced imaging modalities have enhanced the survival rates of breast cancer patients; however 20–30% of early breast cancer patients die of metastatic disease [1]. There is a requisite for identifying specific biomarkers and/or methods through which breast cancer disease progression could be serially monitored and the associated prognosis could be predicted early on. In recent years, liquid biopsy has emerged as a cost-effective and minimally invasive technique and has been identified as a competent method for the diagnosis and screening of biomarkers in breast cancer [2,3,4]. Liquid biopsy or fluid biopsy for cancer diagnosis is a method of analyzing blood samples for the presence of circulating tumor cells (CTCs), circulating cell-free DNA, RNA (ccfDNA, ccfRNA), circulating tumor DNA (ctDNA), exosomes, etc., for monitoring cancer progression. The techniques are minimally invasive and can be utilized for longitudinal serial monitoring of patients. The source of ctDNA in a cancer patient can be from CTCs, primary tumor cells, or metastatic cells lodged at distant metastatic sites in the body and it can be used for detecting genomic patterns and alterations that evolve during treatment. Serum markers CEA or CA15-3 are still in use to follow up disease but they have low specificity [5,6].

The major source of ccfDNA in healthy individuals comprises short fragments of DNA which are <200 bp in length, are most often associated with histone proteins, and hence deemed to be released by the process of apoptosis. In cancer patients, however, necrotic events and endonuclease activities engage in fragmenting the chromatin into longer, nucleosomal units of 180 bp up to 1 kb in length and may be considered as representative of ctDNA. This leads to increasing levels of ccfDNA with elevated levels of longer fragments in the serum or plasma of cancer patients than in healthy controls and it has been reported as an effective blood-based biomarker for various types of cancers [7,8,9].

ctDNA can be discerned from ccfDNA, qualitatively, by evaluating the tumor-specific mutations, methylations, and gene amplifications of these fragments [10]. However, only a limited proportion of patients carry such known mutations; for instance, only 30–40% of breast cancer patients carry PI3KCA mutations [10]. Other known approaches also focus on quantitating total ccfDNA levels using target genes such as GAPDH and β-globin with higher levels observed in malignant disease [10]. However, these results may be influenced by other co-existing conditions such as infection or inflammation which may have a likely effect on the ccfDNA levels. Another well-established methodology is a quantitative approach to assess the copy number of tumor-DNA-associated gene elements. In this regard, LINE elements, a group of self-replicating retrotransposons, are the most used targets accounting for their high copy number, with them occupying nearly 15% of the total genome. Among them, ALU sequences ALU 115 and ALU 247’s copy numbers are associated with the levels of ccfDNA and ctDNA, respectively [11]. The advent of real-time quantitative PCR (q-PCR) has enabled a quick and cost-effective method of detecting the target biomarker ALU elements in ccfDNA from the serum or plasma of cancer patients. The ratio between long and short ccfDNA fragments (DI) has been used as a potential diagnostic marker in multiple cancer types including breast cancer [10,12,13,14]. In this study, we have used liquid biopsy to evaluate prognosis and to monitor disease progression in breast cancer patients by assessing the small and large ALU repetitive element levels. Levels of ALU 247 and DI have been used as a measure to gauge ctDNA levels which are then correlated with various clinico-pathological features in an attempt to identify its utility to predict disease prognosis.

## 2. Materials and Methods

### 2.1. Patient Recruitment

Blood samples (n = 254) were accessed from 167 breast cancer patients at various stages during disease diagnosis and treatment at St. John’s Medical College and Hospital, Bengaluru. One hundred and eleven samples were collected pre-operatively (treatment-naïve) immediately after diagnosis. A figure of 110 were post-operative (collected within 48 h after surgery), 20 were metastatic, and 13 were collected from patients who were in remission at the median follow-up of 47 months. For 88 patients, we had access to paired pre- and post-operative samples. A figure of 66 patients with pre-operative samples and 61 patients with post-operative samples had a long-term follow-up with a median of 72 months. Blood samples collected from metastatic patients either presented with metastasis or were at a median follow-up of 32 months after their primary diagnosis. Written informed consent was obtained from all of the participants and the Institutional Ethics Committee approved the study (IERB No: 167/2019, 62/2008).

### 2.2. Plasma Preparation and DNA Extraction

Five mL of venous blood samples were collected from patients into EDTA tubes and centrifuged for 10 min at 2500 rpm. The plasma was carefully separated and stored in −80 °C until further use. All of the samples were processed within 2 h of collection. DNA extraction was carried out using a QIAamp DNA blood mini kit (QIAGEN, Hilden, Germany) as per the manufacturer’s protocol [15,16,17,18]. The DNA was subjected to Qubit-based quantitation and stored at −20 °C. The cfDNA quantitated ranged from 0.02–2.17 ng/µL.

### 2.3. Quantitative PCR of ALU Repeats

Quantitative PCRs for ALU repeats were performed as described previously by Umetani et al. and Iqbal et al. [3,19]. Briefly, primer sequences were designed to amplify ALU sequences of ALU 115, a 115 bp DNA amplicon, and ALU 247, a 247 bp DNA amplicon. The primer sequences were ALU 115 forward primer-5′-CCTGAGGTCAGGAGTTCGAG-3′ and reverse primer-5′-CCCGAGTAGCTGGGATTACA-3′; and ALU 247 forward primer-5′-GTGGCTCACGCCTGTAATC-3′ and reverse primer-5′-CAGGCTGGAGTGCAGTGG-3′. The ALU 115 amplifies both short and long fragments of the DNA and hence represents the total amount of circulating free DNA (cfDNA) in the plasma that is released from apoptotic cells while ALU 247 amplifies the long fragments of the DNA and hence represents the DNA fragments released from cells because of necrosis (ctDNA). DNA levels were measured by q-PCR in duplicates with a 100 pg template per reaction, using SYBR Green master mix on a LightCycler 480 II (Roche Diagnostics) with a 3 pM concentration of ALU 115 and ALU 247 primers making the reaction volume 10 μL. The DNA copy numbers corresponding to amplified ALU 115 and ALU 247 were evaluated by normalization with known concentrations of genomic DNA by deriving a standard curve, and the DI index was calculated as a ratio of ALU 247 to ALU 115 copy numbers. The annealing sites of ALU115 are within the ALU247 annealing sites hence the ratio of ALU 247 to ALU 115 is called the DI index. This is indicative of the fragmentation pattern of circulating cell-free DNA. The DNA integrity is “1” if template the DNA is not shortened and “0” if the DNA is completely fragmented. A higher DI index indicates higher Alu 247 and thereby an implication of increased ctDNA burden [20].

### 2.4. Next-Gen Sequencing-Primer Synthesis, Library Preparation, and Analysis

The mutations identified in DNA samples from breast cancer patients were verified through database analysis to detect concurrent mutations common in breast cancer patients. Through cBioPortal, the TCGA data for breast cancer of 818 patients were checked for mutations using TP53 and PIK3CA as the query genes. cfDNA samples were sequenced by amplicon sequencing for specific variants of *TP53* and *PIK3CA* using the primers listed in Supplementary Table S1. The primers were designed based on the guidelines provided in the 16S metagenomic sequencing library preparation (15044223 B) manual. Amplification of the target regions in the samples was carried out with a JumpStart kit (P2893-100RXN) by multiplexing all of the primers. The library was prepared by ligating unique barcodes from IDT for Illumina DNA/RNA UD Indexes (20026121). The pooled libraries were sequenced on the NovaSeq 6000 (Illumina, San Diego, CA, USA) platform using 2 × 150 bp chemistry. The reads were quality filtered using Trimmomatic [21], and aligned to the GRCh38 reference using Bowtie2 [22]. The variants were called from the alignment map using multiple pipelines, including freebayes [23] and bcftools [24]. The called variants were analyzed and annotated using wANNOVAR [25] and Ensembl Variant Effect Predictor [26] web-based tools.

### 2.5. Statistical Analysis

Descriptive statistics were used for all of the clinical variables. Differences in estimated levels of DNA fragments and DNA integrity (DI) between the blood samples collected during different stages of disease as mentioned above (in the subsection Patient ecruitment) were assessed using the Mann–Whitney Utest or two-tailed Student’s *t*-test. Kaplan–Meier analysis was used to examine the estimated differences in disease-free survival. Disease-free survival was calculated as the time from the date of first diagnosis to the time when either local recurrence or distant metastasis occurred. Nelson–Aalen analysis, a non-parametric estimator of the cumulative hazard function was also performed to analyze the survival function. Patients without an event or those who had succumbed to non-breast-cancer-related causes were right censored. A log-rank test (Mantel–Cox) was used to compare the survival rates between the groups. Both univariate and multivariate Cox proportional hazard analyses were carried out to validate the prognostic importance of ALU 247 and DI in comparison to other clinico-pathological characteristics. A nomogram was constructed using R software (version 4.1.3) with the package “rms” (version 6.3.0). The performance of the nomogram was assessed by the Harrell’s concordance index (C-index). The C-index has a range from 0.5 to 1.0, with 0.5 indicating random chance and 1.0 considered as perfect discrimination. It was used to assess the accuracy and identification abilities of the predictive factors. To estimate the clinical utility of the nomogram, decision curve analysis using the R package “dcurves” (version 0.3.0) was performed by calculating the net benefits for a range of threshold probabilities. The predicted variables were evaluated for their association with the occurrence of an event (metastasis or death due to metastasis) using multivariate logistic regression models and LASSO regression models. For all of the tests, a *p* value of <0.05 was considered to be statistically significant. All of the statistical analyses were carried out using the software XLSTAT, version 2022.2.1.

## 3. Results

### 3.1. Patient Characteristics

A total of 254 plasma samples isolated from 167 patients in various stages of disease were subjected to cfDNA analysis by q-PCR. A figure of 44% of the samples were treatment-naïve and collected before surgery (pre-operative), 43% were collected within 48 h after surgery (post-operative), and 8% were collected from patients with metastasis at presentation. Samples were also collected from patients in remission at varying time points. Samples collected anytime from 24 months to 56 months were grouped as short-term remission (2%) and those from 56 months to 13 years were grouped as long-term remission (3%) (Supplementary Figure S1). The mean age of all of the patients was 56 ± 13 years. The mean tumor size in the treatment-naïve patients undergoing surgery was 3.5 ± 1.8 cm. The hormone receptor (HR+) and triple-negative (TNBC) subtypes account for 55% and 26% of these tumors, respectively.

### 3.2. Evaluation of ctDNA and the DI Index; ALU 247 Levels Decrease Post-Surgical Intervention and Are Higher in Metastatic Patients

**Figure 1:**
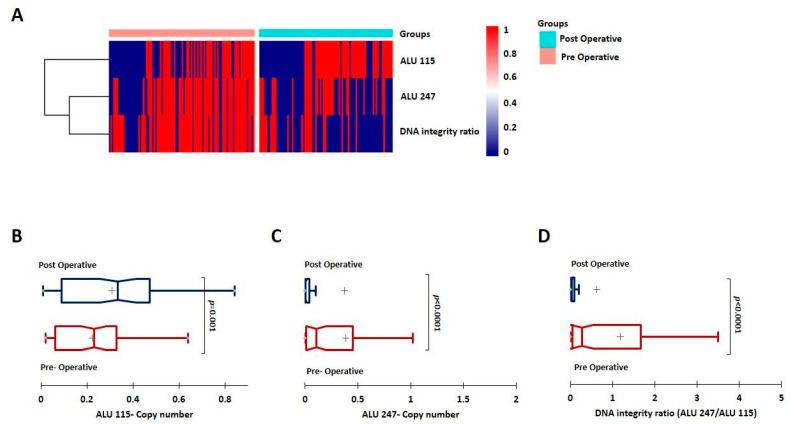
(**A**) Heat map depicting the levels of ALU 115 and ALU 247, and the DI index among post-operative and pre-operative samples. (**B**–**D**) Univariate analysis depicting levels of ALU 115 and ALU 247, and the DI ratio, respectively, among matched/paired post-operative and pre-operative samples (n = 88 each).

After computing the copy numbers of ALU 115 and ALU 247 and the DI index, we first compared them between all of the pre-operative and post-operative samples. ALU 115 levels (representing the total cfDNA) were observed to be higher in the post-operative group, but this difference was not statistically significant (*p* = 0.1). However, the ALU 247 levels (representing the ctDNA) and the DI index were significantly lower in the post-operative groups (*p* < 0.0001) (Figure 1A). ALU 115, ALU 247, and the DI index were also compared between matched/paired pre- and post-operative samples (n = 88). We observed similar trends with ALU 115 being higher in the post-operative group (*p* < 0.05) in these matched samples. As observed earlier, ALU 247 and the DI index were significantly lower in the post-operative samples (*p* < 0.0001) (Figure 1B–D).

**Figure 2:**
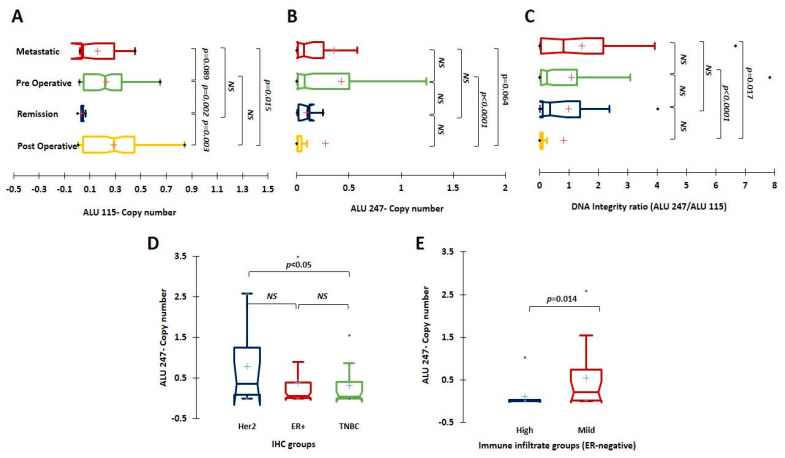
(**A**–**C**) Univariate analysis depicting levels of ALU 115 and ALU 247 and the DI ratio, respectively, among the metastatic (n = 20), pre-operative (n = 111), remission (n = 12), and post-operative samples (n = 108). (**D**) Comparative analysis of ALU 247 levels among IHC groups Her2 (n = 16), ER+ (n = 57), and TNBC (n = 30) and (**E**) between dense/moderate (n = 16) and mild (n = 23) immune infiltrate groups of the ER-negative subtype. *NS*: not significant.

The levels of ALU 115 and ALU 247 and the DI index were then compared between the samples collected during the various stages of disease in different breast cancer patients. ALU 115 levels were found to be significantly lower in patients in remission (combined short-term and long-term) when compared to the pre-operative (*p* = 0.002) and post-operative (*p* = 0.003) groups. They were also lower in the metastatic group when compared to the post-operative samples (*p* = 0.01), and although not statistically significant, they were lower when compared to the pre-operative samples (*p* = 0.08). ALU 247 levels and the DI index were, however, significantly higher in the metastatic group when compared to the post-operative (*p* = 0.06 and *p* = 0.01, respectively) (Figure 2A–C) group.

### 3.3. Implication of Baseline ALU 115, ALU 247, and DI Levels on Prognosis and Association with Tumor Clinico-Pathological Characteristics

To verify the prognostic importance of the DI index, we performed Kaplan–Meier survival analysis on a subset of samples where follow-up information was available. We first examined the pre-operatively collected samples to evaluate the prognostic implication of the ALU markers and the DI index in them. To maximize the specificity, we chose the cut-off at the third quartile (0.492 for ALU 115, 0.042 for ALU 247, and 0.08 for the DI index) and divided the samples into high and low groups based on the cut-off values. In the 50 samples where the analysis was performed, we did not observe any statistically significant difference in survival based on stratification by ALU 115, ALU 247, or DI (*p* > 0.05). To further examine the association of ALU levels and the DNA index with tumor clinico-pathological characteristics, we analyzed the known clinical features associated with pre-operatively collected tumors (treatment-naïve) and performed a correlative analysis. The pre-operative samples were stratified based on IHC classification into HR+; n = 57, HER2+; n = 16, and the TNBC; n = 30. The DI index and ALU 247 levels were significantly higher in the HER2 subtype when compared to the TNBC (*p* < 0.05) and relatively higher than the HR+ subtypes although not statistically significant (Figure 2D, Supplementary Figure S2). There was no significant difference in the distribution of ALU 115 levels across the various subtypes. ALU 115 levels, however, increased with higher tumor stage and size, but lower levels were associated with a higher Ki67 index (*p* < 0.05) (Table 1).

We did not observe any significant associations with other clinico-pathological features such as tumor size, tumor grade, or stage or lymph node status. (Table 1 and Supplementary Table S2). Although, the DI index was found to be higher in tumors with a higher grade and stage, the observation was not statistically significant (*p* > 0.05). The samples were then stratified based on ER-positivity and the same analysis was performed between ER-negative and ER-positive tumors. We did not observe any associations with any of the clinico-pathological features in the ER-positive subtype. However, we observed that the DI index and ALU 247 levels were positively associated with a mild immune infiltrate in the ER-negative subtype (n = 39). The tumors were separated into groups (dense/moderate and Mild) based on the immune filtrate as recorded in the pathological reports. Tumors with mild immune infiltrate were associated with a significantly higher DI index (*p* = 0.01) and higher amounts of ctDNA as represented by ALU 247 (*p* = 0.01) (Figure 2E, Supplementary Figure S3).

**Figure 3:**
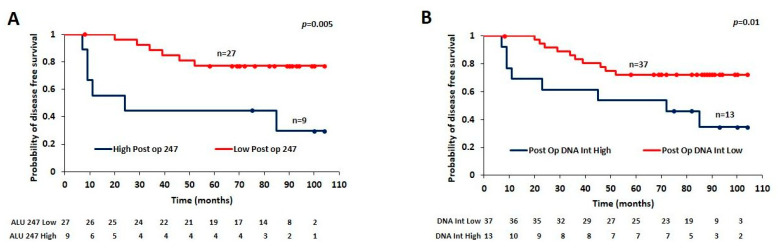
(**A**): disease-free survival between ALU 247 low (n = 27) and ALU 247 high (n = 9) samples and (**B**): between DI low (n = 37) and DI high (n = 13) samples.

We then evaluated the prognostic implication of ALU 115, ALU 247, or DI on post-operatively collected samples by Kaplan–Meier analysis. The third quartile cut-off was used again for all of the analysis (0.498 for ALU 115, 0.03 for ALU 247, and 0.07 for the DI index) and the samples were divided into high and low groups based on the cut-off. ALU 115 did not demonstrate any statistically significant separation based on survival (*p* = 0.1). However, stratification of the samples by both ALU 247 levels (log rank *p* = 0.005) and the DI index (log rank *p* = 0.01) produced significant separation of the groups based on disease-free survival. The disease-free survival at the 6-year mean follow-up dropped from 84 months in ALU 247 low samples to 44 months in ALU 247 high samples and from 82 months in DI low samples to 58 months in DI high tumors (Figure 3A,B).

### 3.4. Detection of Mutations from ctDNA by NGS

To confirm if the DNA fragments evaluated by ALU 247 were derived from tumor DNA, we examined them for commonly observed mutations in breast cancer using the NGS approach. An NGS-based short panel analysis was carried out on 19 random samples with high ALU 247 levels as described in the Section 2 to detect concurrent mutations commonly observed in *TP53* and *PIK3CA* among breast cancer patients. The PIK3CA variant [179234250-251 (TG > CT)] was detected in 17/19 of the samples and the TP53 variant [7673432-434 (TGG > GGC)] was detected in 4/19 of the blood samples tested. This result confirmed that DNA identified by higher ALU 247 levels is likely to represent the breast tumor DNA in circulation.

### 3.5. Utility of Estimating Post-Surgical ALU 247 Levels for Predicting Prognosis

Encouraged by the observation that ALU 247 levels and DI estimated post-operatively identified patients with poor prognosis based on disease-free survival analysis, we wanted to further probe the clinical utility of these factors for predicting disease progression. The prognostic values of ALU 247 and DI were further verified using univariate Cox proportional hazard analysis (Table 2).

The risk associated with high ALU 247 and DI levels was estimated with other clinical variables including age, tumor size, tumor grade, and lymph node status. ALU 247 levels and lymph node status emerged as highly significant independent variables after univariate analysis, while the DI, age, tumor size, and tumor grade were of no significance—ALU 247: HR 1.302 (95% CI 1.074–1.578, *p* = 0.007); lymph node status: HR 9.441 (95% CI 2.026 to 44.003, *p* = 0.004) DI: HR 0.856 (95% CI 0.659–1.110, *p* = 0.24). Since only ALU 247 and not DI emerged as a significant variable, further analysis was performed with ALU 247. Nelson–Aalen analysis, a non-parametric estimator of the cumulative hazard function also showed significant stratification of the ALU 247 high and low groups based on disease-free survival (log-rank; *p* = 0.005 and Wilcoxon; *p* = 0.004). In multivariate analyses, with the other prognostic markers, ALU 247 levels measured post surgically evolved as an independent prognostic factor [ALU 247: HR 1.3 (95% CI 1.047 to 1.613, *p* = 0.017)] (Table 2).

**Figure 4:**
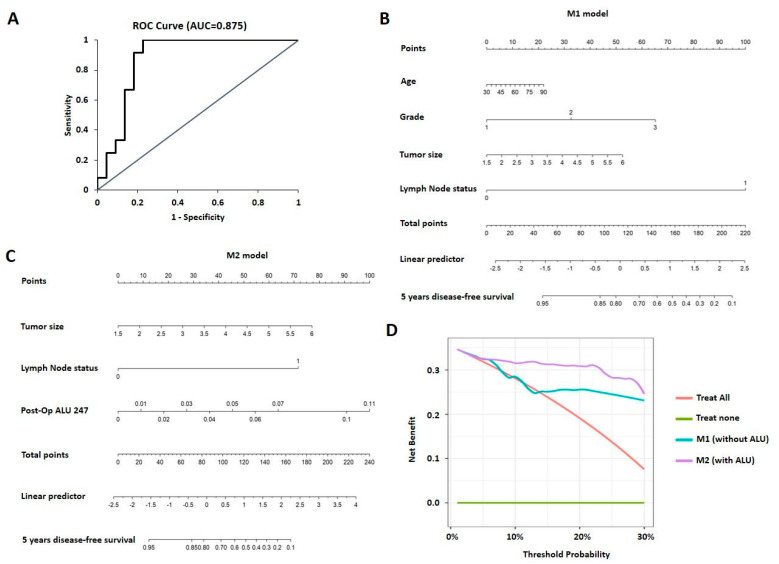
(**A**) ROC curve for ALU 247 with other parameters with AUC analysis derived by logistic regression. (**B**) Nomogram analysis with the M1 model depicting disease-free survival based on conventional parameters. (**C**) Nomogram analysis with the M2 model depicting disease-free survival based on conventional parameters along with post-operative ALU 247 levels. (**D**) Decision curve analysis derived for the nomogram M1 and M2 depicting the net benefit of the model across different threshold probabilities.

A Lasso regression model was then used to assess the weight of ALU 247 towards determining the occurrence of an event (metastasis or death due to disease) along with other clinical parameters of age, tumor size, tumor grade, and lymph node status. The model predicted ALU 247 levels to be the most influential factor with the highest variable importance score and co-efficient value of 1.789 (Supplementary Figure S4) along with other clinical parameters. The Lasso regression model by including ALU 247 gave an improved R^2^ of 0.36 and a lower root mean square error (RMSE) of 0.38 when compared to the model with all of the other conventional parameters of age, tumor size, tumor grade, and lymph node status excluding ALU 247 (R^2^ of 0.30 and a RMSE of 0.399). Cross-validation confirmed that the model where ALU 247 is combined with conventional parameters produced a lower lambda value of 0.07 (when compared to 0.087 for the conventional model) and hence, it emerges as the more robust performing model. Furthermore, a logistic regression model and analysis performed by including ALU 247 with other parameters improves the sensitivity of the assay by 8% and renders a higher AUC of 0.875 (Figure 4A).

### 3.6. Nomogram and Decision Curve Analysis for Prediction of Prognosis

Next, we constructed various nomograms using conventional parameters of age, tumor size, lymph node status, and tumor grade. Different models were constructed using clinical parameters alone (M1) and using two clinical parameters along with post-operative ALU 247 which exhibited significant differences in the multivariate analysis: tumor size and lymph node status (M2). Each factor was ascribed a weighted point, and the total points indicated the risk of disease-free survival. The variables used as factors and the final nomogram model are presented in Figure 4B,C. To evaluate the predictive accuracy of the nomogram prediction system, the C-index was calculated and validated between the two models. For the nomogram model built with the conventional parameters (M1), the C-index was 0.772. For the model M2 after inclusion of post-operative ALU 247, the C-index improved to 0.823 which indicates a better ability to assign patients to an accurate risk stratification and a higher C-index of more than 0.8 suggesting a stronger model. A decision curve analysis was then derived to assess the net benefit of the model across different threshold probabilities. The decision curve analysis for the two models is presented in Figure 4D. The analysis predicted that the model integrating ALU 247 along with other clinical parameters was of value at a threshold of 25% at 5 years with a net benefit of 0.039. These results suggest that ALU 247 levels measured post-operatively can be used as a tool to predict prognosis in breast cancer. This adds value to the already employed clinical parameters that are routinely used to assess the risk of progressive disease after surgical intervention.

## 4. Discussion

Biopsy is still deemed the right tool for confirmation of cancer diagnosis but has apparent deficiencies that include an inability to capture the dynamic changes incurred by anti-cancer therapies and other genomic insults that occur during treatment. In addition, serial biopsies may not always be feasible in the clinical setting. The use of circulating biomarkers from the peripheral blood as a liquid biopsy tool has been subjected to exploration over the past several years due to the apparent advantage of being minimally invasive. Analysis of circulating nucleic acids and CTCs provides a diversity of data including genomic changes, treatment response, and prognosis which can not only be used for detection but also to monitor disease progression. The ratio between the long and short cfDNA fragments (DI) has been shown to be a potential diagnostic marker in multiple cancer types as described below. DI was found to be significantly increased in patients with colorectal cancer [27,28] and was found to be correlated with poor prognosis in these patients [29]. In non-small cell lung cancer, higher levels of ALU 247 and 115 were associated with higher stages and metastatic disease [30]. High levels of ctDNA are associated with a more aggressive, potentially resistant disease and have been detected both in the early and later stages of breast cancer [31,32,33]. We have estimated the levels of ALU 115 and ALU 247 and the DI index in breast cancer patients at various stages of disease and observed a significant reduction in ALU 247 values (representing the ctDNA) and the DI index in the post-operative period. This result could be in resonance and reflect effective surgical removal of the primary tumor. A previous study by Iqbal et al. reported similar results whereby they observed a significant decline in DI after surgery while this change was not observed for ALU 247 [3]. Elhelaly et al. and Hassan et al. also recently reported similar findings by assessing cell-free DNA quantity post-surgery, and there was a drop in DNA concentrations after elimination of macroscopic tumor burden [34,35].

We also observed a significant reduction in the total cell-free DNA levels identified by ALU 115 and a marked increase in the levels of ALU 247 and the DI index in the metastatic patients when compared to the post-operative samples. The increase in the baseline DI in relapsed patients compared to patients who were free of disease was also reported by Iqbal et al. [3]. In another study, using LINE-1 and ALU markers, baseline cfDNA and DI were shown to be independent prognostic markers in metastatic breast cancer patients [36]. The ability of DI alone to detect metastasis has been contradictory, however, as another report observed that evaluating levels of both plasma ALU 247 and ALU 115 was useful in identifying patients who developed metastasis in breast cancer, but cfDNA integrity failed to do this [37]. Changes in sample processing, experimental procedures, and methodologies involved in DNA extraction could have contributed to these variations. To overcome this, we employed the ALU primer pairs that have been widely used by other groups and standard methodologies to confirm [2,14,28,37,38,39] the authenticity of the results we have obtained.

Further evaluation of the prognostic implication of the baseline (pre-operative levels) ALU and the DI indices did not yield statistically significant difference in survival in our study, as opposed to earlier reports in a Finnish cohort where DI was measured using a fluorometer and another meta-analysis of eight different studies which performed ctDNA gene variation detection [4,32]. In these studies, pre-operative ctDNA levels were significantly associated with shorter disease-free survival. Further correlation of ALU levels and DI with tumor clinico-pathological characteristics showed that DI was significantly higher in the HER2 subtype. This observation is in accordance with Hussein et al. [37] whereby they observed a marked increase in cfDNA integrity in breast cancer patients with HER2 amplification which may be attributed to the increased proliferation associated with the HER2 subtype.

We did not observe any associations with other clinico-pathological features such as tumor size, grade, and stage or lymph node status in accordance with Hussein et al.; however, it is noteworthy that some studies have reported a positive correlation of the DI index with TNM staging [33] to the size of invasive cancers and the presence of lymphovascular invasion or lymph node status [19]. Interestingly, very few studies have recorded a correlation between immunotherapy resistance and ctDNA levels in breast cancer. The presence of high microsatellite instability detected in ctDNA is reported to be associated with a good response to immunotherapy, and assessment of dynamic levels of ctDNA during pembrolizumab treatment revealed that a rise in ctDNA levels during treatment correlated with a progressive disease and poor survival [40,41]. We report for the first time a positive correlation between milder immune infiltration and ALU 247 and DI levels in ER-negative tumors. A milder immune infiltrate is associated with poorer prognosis in the ER-negative subtype of breast cancer [42], and higher levels of ctDNA as measured by our assay may aid in stratifying tumors based on immune infiltrate. This observation needs additional validation on a larger set of tumors and if proven useful, it may profoundly benefit in identifying tumors that may qualify for immunotherapy especially in the metastatic setting.

Although our results support the prognostic potential of the DI index, the multivariate survival analysis DI index failed to emerge as an independent prognostic factor. The DI index is computed as a relative proportion and is contingent on ALU 115 levels. ALU 115 represents cfDNA and the levels of the same may vary depending on multiple physiological factors including inflammation and other co-morbidities. This could partially explain why the DI index failed to emerge as an independent prognostic factor. However, ALU 247 emerged as an independent prognostic factor with Cox regression survival analysis as larger fragments are likely to be derived from tumor DNA, which was further confirmed by mutations observed by sequencing. This prompted us to explore the possibility of inclusion of ALU 247 along with other conventional parameters to assess disease progression in breast cancer. Inclusion of ALU 247 levels in the lasso and multivariate logistic regression model improved the predictive performance of the model. Cross-validation confirmed that a model where ALU 247 was combined with conventional parameters provided a more robust performance and a lesser mean squared error. Nomogram-based decision curve analysis suggests that the combined use of established clinico-pathological features and liquid biopsy could help to discriminate breast cancer patients prone to poor disease-free survival during the immediate post-operative period.

This study has several limitations including its retrospective study design and the limited number of samples with follow-up data used for survival and model construction. The availability of long-term follow-up information in only a small number of patients was a disadvantage. Nucleic acid extraction from blood samples poses significant analytical and experimental challenges, and despite having accessed stored blood samples, we followed strict quality control measures and protocols to eliminate such hitches. To prevent genomic DNA contamination, we employed plasma as the source of ctDNA which is in line with other studies that have been performed [43]. Though our results indicate that the levels of ALU 247 are likely to be derived from tumor DNA, this validation was carried out for a limited number of mutations in a small subset of samples. This needs further validation with gold standard methods such as analysis of a larger panel of frequently mutated loci of cancer-associated genes through NGS. Nevertheless, our effort to build a nomogram for clinical utility using the DNA fragment estimated by the ALU-247 is different from previous reports, thus adding novelty and clinical relevance to existing data on cell-free DNA detection in breast cancer.

## 5. Conclusions

Our observations support the utility of evaluating large circulating DNA fragments in the plasma of breast cancer patients for prognosis and monitoring disease progression. Together with conventional clinico-pathological features, this non-invasive and easily reproducible liquid biopsy assay can be used to identify patients prone to poor prognosis. The techniques employed in our study are rapid, low-cost, and accessible diagnostics and can be deployed as supplementary measures to the consensus-based gold standard sequencing approaches employed in liquid biopsy.

## Figures and Tables

**Table 1:** Association between ALU 115, ALU 247, and DNA integrity ratio with various clinico-pathological features of primary tumors from n = 132 breast cancer patients.

Sl No.	Parameter (n)	Category	Rel. Frequency/Category (%)	qPCR ALU115 Mean	*p* Value (Mann–Whitney)	qPCR ALU247 Mean	*p* Value (Mann–Whitney)	qPCR DNA Integrity Ratio Mean	*p* Value (Mann–Whitney)
1	Age (129)	<50	29	0.227	0.837	0.611	0.360	1.485	0.464
		>50	71	0.233		0.364		0.966	
2	Age at Menarche (118)	Early	14	0.197	0.259	0.402	0.453	1.029	0.462
		Late	86	0.252		0.490		1.175	
3	Menopausal status (129)	Pre-	26	0.225	0.912	0.554	0.520	1.242	0.864
		Post-	74	0.233		0.398		1.071	
4	Breast side (129)	Left	55	0.227	0.846	0.488	0.679	1.304	0.436
		Right	43	0.234		0.350		0.861	
5	Diabetic (109)	Yes	33	0.240	0.585	0.295	0.087	0.733	0.053
		No	67	0.253		0.597		1.477	
7	Hypertension (114)	Yes	48	0.250	0.986	0.433	0.313	1.097	0.372
		No	52	0.241		0.518		1.376	
8	Tumor grade (128)	1	19	0.228	1 v/s 2–0.641	0.155	1 v/s 2–0.704	0.464	1 v/s 2–0.494
		2	55	0.244	1 v/s 3–0.788	0.561	1 v/s 3–0.952	1.396	1 v/s 3–0.886
		3	21	0.195	2 v/s 3–0.353	0.300	2 v/s 3–0.623	0.873	2 v/s 3–0.577
9	Tumor size (127)	<3cm	40	0.185	**0.048**	0.437	0.409	1.094	0.639
		>3cm	60	0.250		0.414		1.102	
10	Lymph node status (125)	Positive	42	0.249	0.407	0.394	0.749	0.964	0.348
		Negative	58	0.221		0.471		1.202	
11	Lymphovascular invasion (108)	Present	40	0.244	0.597	0.376	0.844	0.953	0.526
		Absent	60	0.230		0.489		1.273	
12	Tumor stage (117)				1 v/s 0–0.953		1 v/s 0–0.953		1 v/s 0–0.961
		0	3	0.170	2 v/s 0–0.344	0.048	2 v/s 0–0.210	0.287	2 v/s 0–0.513
		1	15	0.161	3 v/s 0–0.166	0.435	3 v/s 0–0.439	1.126	3 v/s 0–0.903
		2	50	0.238	2 v/s 1–0.121	0.515	2 v/s 1–0.141	1.340	2 v/s 1–0.304
		3	32	0.291	3 v/s 1–**0.025**	0.433	3 v/s 1–0.328	1.038	3 v/s 1–0.644
					3 v/s 2–0.209		3 v/s 2–0.828		3 v/s 2–0.387
13	Ki-67 index (91)	<15	40	0.279	**0.044**	0.531	0.218	1.290	0.890
		>15	60	0.188		0.408		1.153	
14	Immune infiltrate (120)	Mild	43	0.252	0.309	0.572	0.263	1.456	0.098
		High	43	0.218		0.374		0.980	

The bold highlighted the significant (*p* < 0.05) *p* values.

**Table 2:** Univariate and multivariate Cox proportional hazard analysis carried out to validate the prognostic importance of ALU 247 in comparison to other clinico-pathological characteristics.

	All; n = 36			
	Univariate		Multivariate	
	HR (95% CI)	*p*-Value	HR (95% CI)	*p*-Value
Age	1.006 (0.965–1.05)	0.76	1.013 (0.972–1.056)	0.537
T-size	1.416 (0.911–2.203)	0.12	1.604 (0.990–2.601)	**0.05**
Lymph Node status				
Negative	Reference			
Positive	9.441 (2.026–44.003)	**0.004**	8.073 (1.611–40.449)	**0.01**
Grade				
I and II	Reference			
III	0.808 (0.175–3.735)	0.78	1.347 (0.248–7.300)	0.73
Post-Operative ALU 247	1.302 (1.074–1.578)	**0.007**	1.3 (1.047–1.613)	**0.017**

The bold highlighted the significant (*p* < 0.05) *p* values.

## Data Availability

Not applicable.

## Supplementary Materials

The following supporting information can be downloaded at: https://www.mdpi.com/article/10.3390/cancers15041054/s1. Figure S1: Proportion percentage of various disease groups subjected to cfDNA analysis in the study; Figure S2: Univariate analysis depicting DNA integrity ratio between the IHC subtypes; Figure S3: Univariate analysis depicting DNA integrity ratio between high and mild immune infiltrate groups in the ER-negative subtype; Figure S4: Lasso regression model analysis performed with ALU 247 and other clinical parameters. The graph represents the weightage the lasso model has assigned to the parameters in determining metastasis or death due to metastasis. Post-Op ALU 247 has the highest weightage; Table S1: Primers for TP53 and PIK3CA, designed based on the guideline provided in 16S metagenomic sequencing library preparation (15044223 B) manual for NGS; Table S2: Clinical characteristics of patients: Clinico-pathological characteristics of (n = 132) patients with primary tumors.
