# Supplementary material for: Estimation of ALU Repetitive Elements in Plasma as a Cost-Effective Liquid Biopsy Tool for Disease Prognosis in Breast Cancer
Source: Cancers (Basel). 2023 Feb 7;15(4):1054. doi: 10.3390/cancers15041054 (PMC9953974; doi:10.3390/cancers15041054)
Supplement: Supplementary file 1 [file cancers-15-01054-s001.zip › cancers-2139161-supplementary.pdf]

Supplementary figures

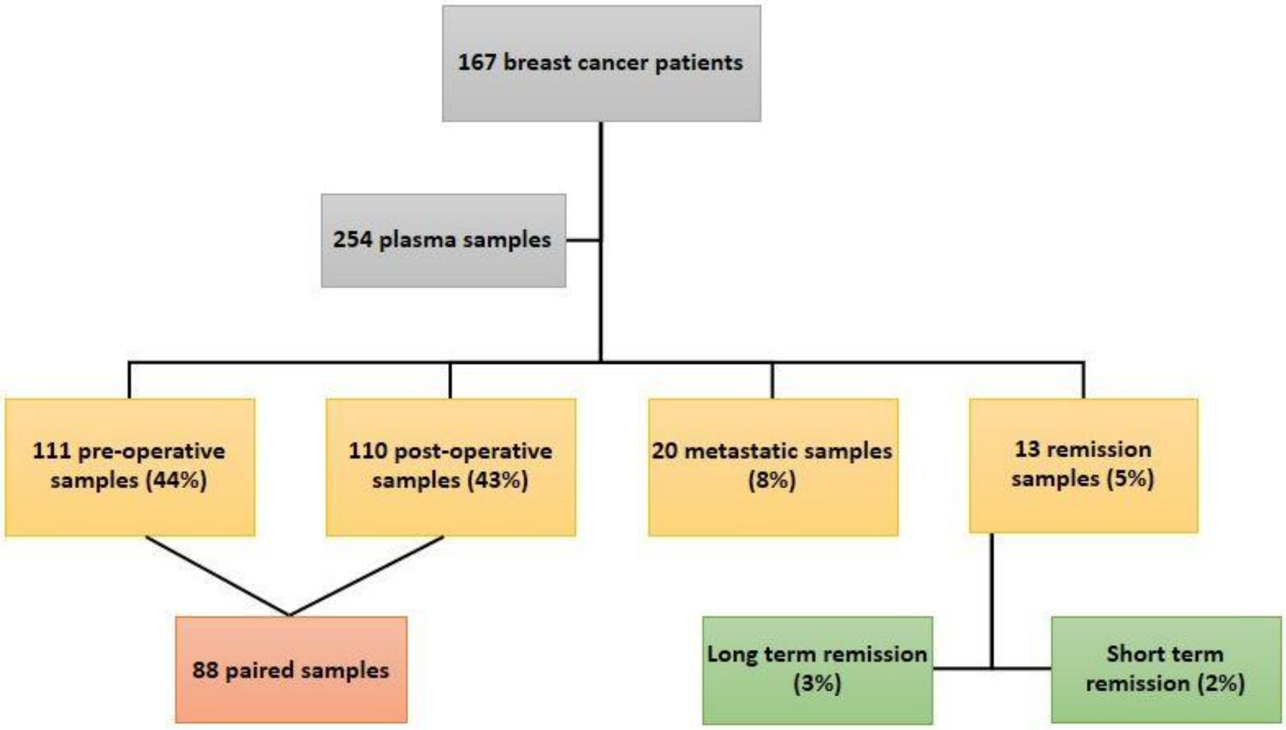

Figure S1. Proportion percentage of various disease groups subjected to cfDNA analysis in the study.

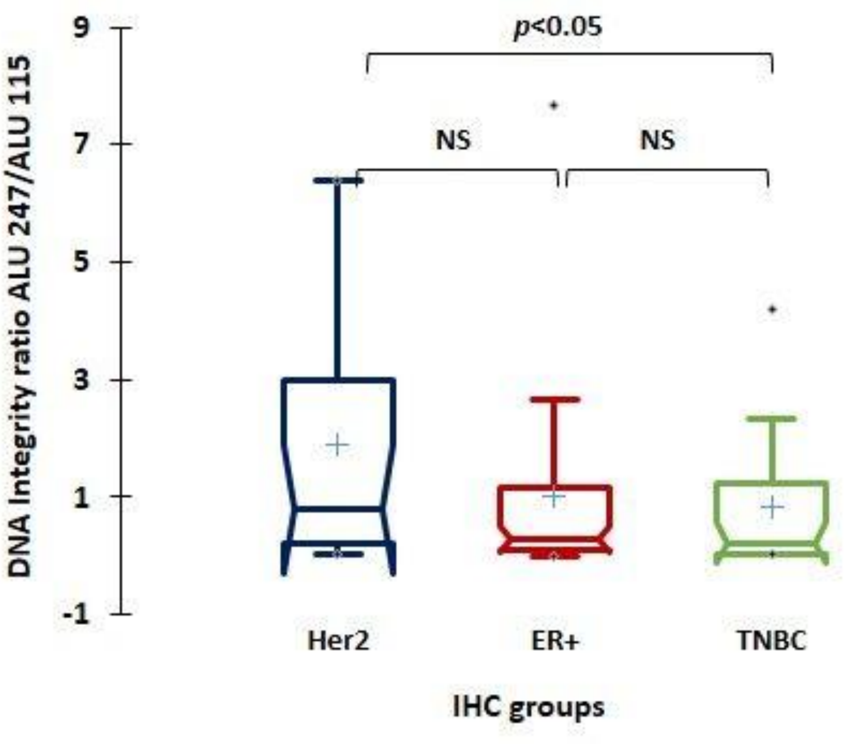

Figure S2: Univariate analysis depicting DNA integrity ratio between the IHC subtypes

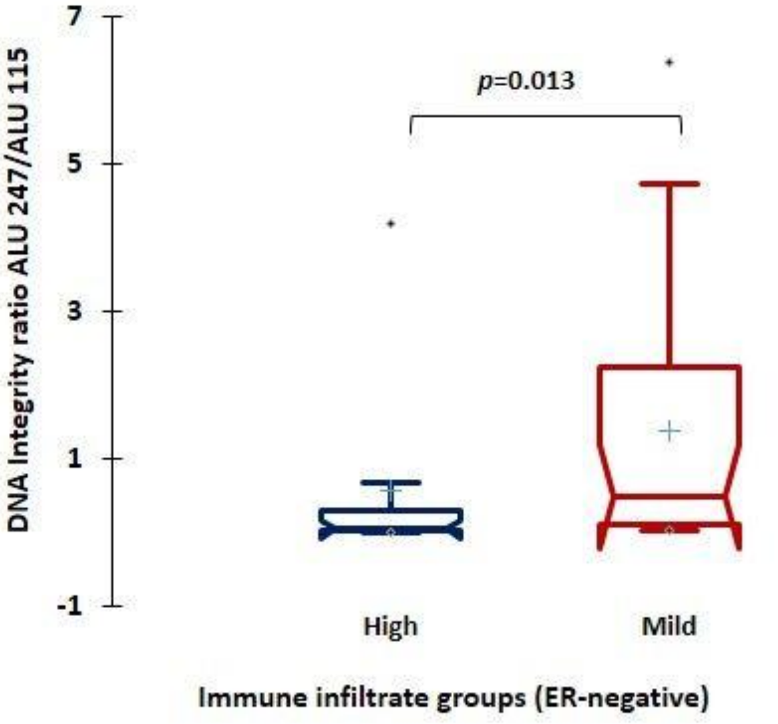

Figure S3: Univariate analysis depicting DNA integrity ratio between high and mild immune infiltrate groups in the ER-negative subtype

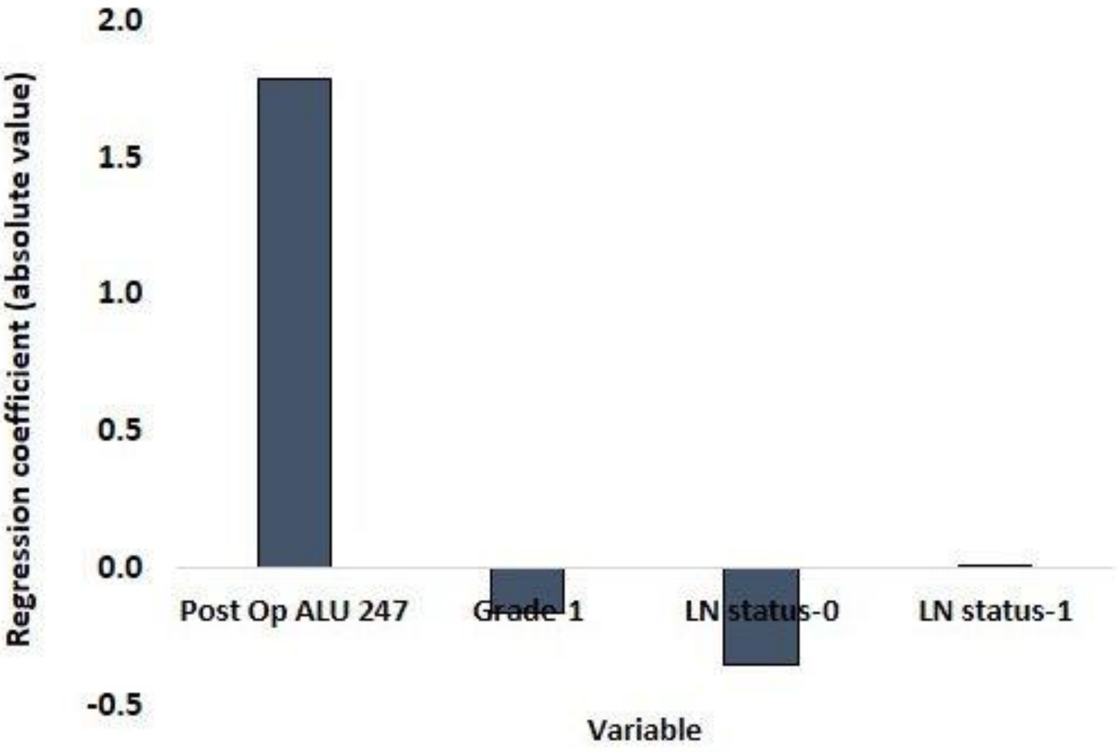

Figure S4: Lasso regression model analysis performed with ALU 247 and other clinical parameters. The graph represents the weightage the lasso model has assigned to the parameters in determining metastasis or death due to metastasis. Post-Op ALU 247 has the highest weightage.

Table S1. Primers for TP53 and PIK3CA, designed based on the guideline provided in 16S metagenomic sequencing library preparation (15044223 B) manual for NGS.

Primer Name	Sequences 5′ to 3′	Length
TP53 EXON 5 FP	TCGTCGGCAGCGTCAGATGTGTATAAGAGAC AGACTCTGTCTCCTTCCTCTTCCT	55
TP53 EXON 5 RP	GTCTCGTGGGCTCGGAGATGTGTATAAGAGA CAGAACCAGCCCTGTCGTCTCTTC	54
TP53 EXON 6 FP	TCGTCGGCAGCGTCAGATGTGTATAAGAGAC AGAGGGTCCCCAGGCCTCT	50
TP53 EXON 6 RP	GTCTCGTGGGCTCGGAGATGTGTATAAGAGA CAGAGCCCCCCTACTGCTCAC	52
TP53 EXON 7 FP	TCGTCGGCAGCGTCAGATGTGTATAAGAGAC AGTGGCCTCATCTTGGGCCTG	52
TP53 EXON 7 RP	GTCTCGTGGGCTCGGAGATGTGTATAGAGAC AGGGTCAGAGGCAAGCAGAGG	
TP53 EXON 8 FP	TCGTCGGCAGCGTCAGATGTGTATAAGAGAC AGGGGAGTAGATGGAGCCTGGT	53
TP53 EXON 8 RP	GTCTCGTGGGCTCGGAGATGTGTATAAGAGA CAGCCGCTTCTTGTCCTGCTTGC	54
TP53 EXON 9 FP	TCGTCGGCAGCGTCAGATGTGTATAAGAGAC AGGGAGGAGACCAAGGGTGCA	52
TP53 EXON 9 RP	GTCTCGTGGGCTCGGAGATGTGTATAAGAGA CAGGGCAAATGCCCCAATTGCAGG	55
PIK3CA FP	TCGTCGGCAGCGTCAGATGTGTATAAGAGAC AGCTGAGCAAGAGGCTTTGGAGTA	55
PIK3CA RP	GTCTCGTGGGCTCGGAGATGTGTATAAGAGA CAGGTGCAATTCCTATGCAATCGGTCT	58

Table S2. Clinical characteristics of patients: Clinico-pathological characteristics of (n=132) patients with primary tumors

	**n = 132 patients (%)**
**Age**	
Mean (Yrs)	56
Median (Yrs)	56
**Tumor Size**	
Mean (cm)	3.6
Median (cm)	3
**Stage**	
I	21 (18)
II	59(50)
III	37 (32)
**Grade**	
0&1	24 (19)
2	71 (55)
3	27 (21)
NA	6 (5)
**Menopausal status**	
Pre	33 (26)
Post	96 (74)
**Lymph node status**	
Positive	53 (42)
Negative	72 (58)
**Estrogen Receptor**	
Positive	77 (61)
Negative	50 (39)
**HER2**	
Positive	19 (15)
Negative	103 (81)
Equivocal	5 (4)
